# Machine learning for improving high‐dimensional proxy confounder adjustment in healthcare database studies: An overview of the current literature

**DOI:** 10.1002/pds.5500

**Published:** 2022-07-05

**Authors:** Richard Wyss, Chen Yanover, Tal El‐Hay, Dimitri Bennett, Robert W. Platt, Andrew R. Zullo, Grammati Sari, Xuerong Wen, Yizhou Ye, Hongbo Yuan, Mugdha Gokhale, Elisabetta Patorno, Kueiyu Joshua Lin

**Affiliations:** ^1^ Division of Pharmacoepidemiology and Pharmacoeconomics, Brigham and Women's Hospital Harvard Medical School Boston Massachusetts USA; ^2^ KI Research Institute Kfar Malal Israel; ^3^ IBM Research–Haifa Labs Haifa Israel; ^4^ Global Evidence and Outcomes Takeda Pharmaceutical Company Ltd. Cambridge Massachusetts USA; ^5^ Department of Epidemiology, Biostatistics, and Occupational Health McGIl University Montreal Canada; ^6^ Department of Health Services, Policy, and Practice Brown University School of Public Health and Center of Innovation in Long‐Term Services and Supports, Providence Veterans Affairs Medical Center Providence Rhode Island USA; ^7^ Real World Evidence Strategy Lead Visible Analytics Ltd Oxford UK; ^8^ Health Outcomes, Pharmacy Practice, College of Pharmacy University of Rhode Island Kingston Rhode Island USA; ^9^ Global Epidemiology AbbVie Inc. Illinois USA; ^10^ Canadian Agency for Drugs and Technologies in Health Ottawa Canada; ^11^ Pharmacoepidemiology Center for Observational and Real‐world Evidence Merck Pennsylvania USA; ^12^ Department of Medicine Massachusetts General Hospital and Harvard Medical School Boston Massachusetts USA

**Keywords:** causal inference, confounding, machine learning

## Abstract

**Purpose:**

Supplementing investigator‐specified variables with large numbers of empirically identified features that collectively serve as ‘proxies’ for unspecified or unmeasured factors can often improve confounding control in studies utilizing administrative healthcare databases. Consequently, there has been a recent focus on the development of data‐driven methods for high‐dimensional proxy confounder adjustment in pharmacoepidemiologic research. In this paper, we survey current approaches and recent advancements for high‐dimensional proxy confounder adjustment in healthcare database studies.

**Methods:**

We discuss considerations underpinning three areas for high‐dimensional proxy confounder adjustment: (1) feature generation—transforming raw data into covariates (or features) to be used for proxy adjustment; (2) covariate prioritization, selection, and adjustment; and (3) diagnostic assessment. We discuss challenges and avenues of future development within each area.

**Results:**

There is a large literature on methods for high‐dimensional confounder prioritization/selection, but relatively little has been written on best practices for feature generation and diagnostic assessment. Consequently, these areas have particular limitations and challenges.

**Conclusions:**

There is a growing body of evidence showing that machine‐learning algorithms for high‐dimensional proxy‐confounder adjustment can supplement investigator‐specified variables to improve confounding control compared to adjustment based on investigator‐specified variables alone. However, more research is needed on best practices for feature generation and diagnostic assessment when applying methods for high‐dimensional proxy confounder adjustment in pharmacoepidemiologic studies.

**Key Points**

- To improve confounding control in healthcare database studies, data‐driven algorithms can be used to leverage the large volume of information in healthcare databases to generate and identify features that indirectly capture information on unmeasured or unspecified confounding factors (proxy confounders).
- Three areas to consider for data‐driven high‐dimensional proxy confounder adjustment include: (1) feature generation—transforming raw data into covariates (or features) to be used for proxy adjustment; (2) covariate prioritization, selection and adjustment; and (3) diagnostic assessment.
- There is a large literature on methods for high‐dimensional confounder prioritization/selection, but relatively little has been written on best practices for feature generation and diagnostic assessment. Consequently, these areas have particular limitations and challenges when applying machine learning algorithms for high‐dimensional proxy confounder adjustment.

**Plain Language Summary**

A fundamental obstacle in studies that utilize administrative healthcare databases is unmeasured confounding bias stemming from nonrandomized treatment choices and poorly measured comorbidities. Failing to adjust for important confounding factors can make it difficult to differentiate between outcomes that are due to drug effects or a result of the underlying conditions for which the drug was prescribed. Traditional approaches for confounding adjustment rely on the investigator to specify all factors that may confound a causal treatment‐outcome association. However, adjustment based on investigator‐specified covariates alone is often inadequate because some important confounding factors are often unknown. Furthermore, because routine‐care databases are not collected for research purposes, many important confounding factors are not directly measured in these data sources. To reduce bias caused by unspecified or unmeasured confounders, many studies have proposed using data‐driven algorithms to identify and control for large numbers of variables that are indirectly associated with unmeasured (or unspecified) confounders ('proxy' confounders). Here, discuss various aspects of high‐dimensional proxy confounder adjustment and give an overview of the current literature. We give particular focus on methods that have been impactful in pharmacoepidemiology research.

## 1 INTRODUCTION

Routinely‐collected healthcare data are increasingly being used to generate real‐world evidence (RWE) to inform decision making in clinical practice, drug development, and health policy.^1^ However, unmeasured confounding from non‐randomized treatment allocation and poorly measured information on comorbidities, disease progression, and disease severity remains a fundamental obstacle to effectively utilizing these data sources for RWE generation.^2^ Statistical methods should therefore be used to extract the maximum possible information on confounding from the data to minimize the effects of unmeasured confounding so that accurate comparative estimates of treatments' effectiveness and safety can be obtained. Approaches to mitigate confounding bias would ideally be based on causal diagrams and expert knowledge for confounder selection.^3^ However, adjustment based on researcher‐specified variables alone is not always adequate because some confounders are either unknown to researchers or not directly measured in these data sources.

**FIGURE 1:**
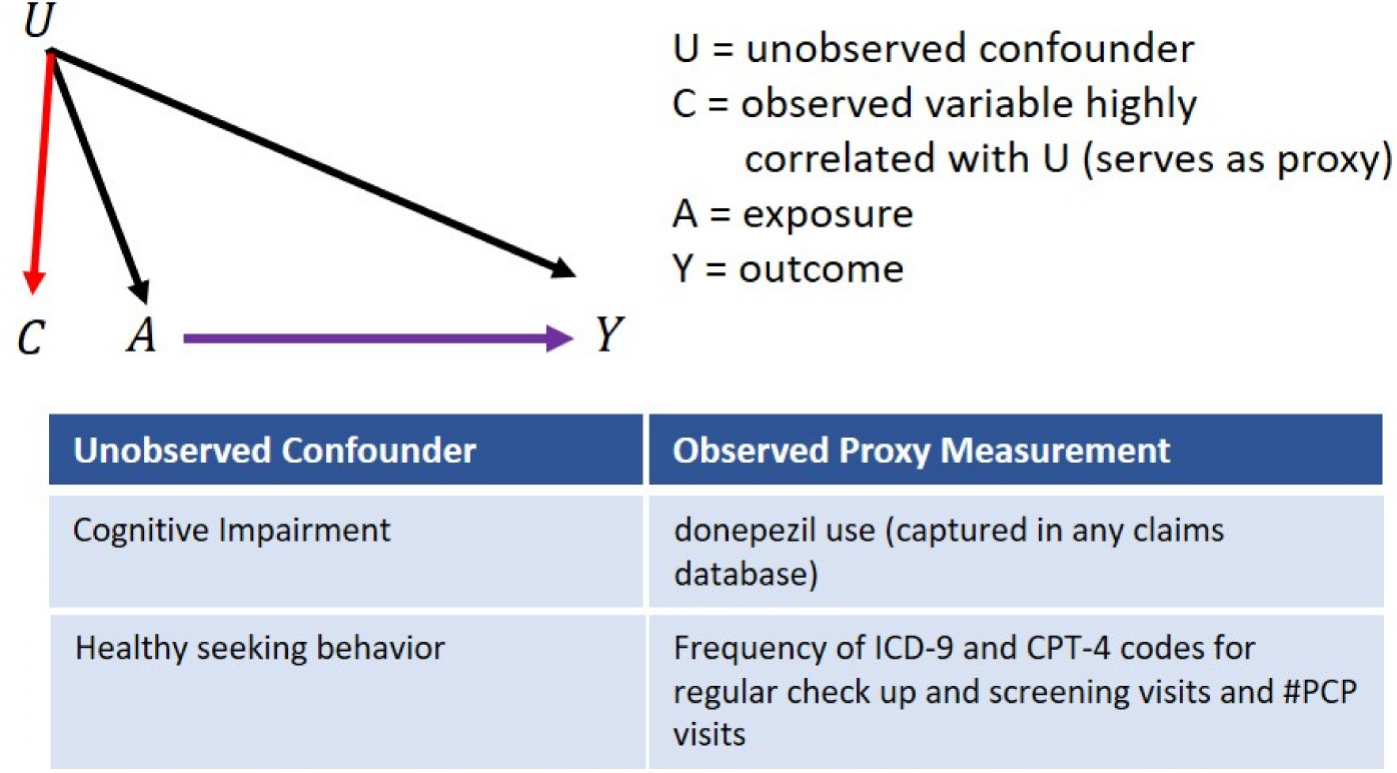
Illustration and examples for ‘proxy confounder’ adjustment.

To improve confounding control in healthcare database studies, data‐driven algorithms can be used to leverage the large volume of information in these data sources to generate and identify features that indirectly capture information on unmeasured or unspecified confounding factors (proxy confounders).^4^ Proxy confounder adjustment is based on the concept that unmeasured confounding can be mitigated by adjusting for large numbers of variables that collectively serve as proxies for unobserved factors.^5^ For example, donepezil use (captured in any claims database) could be used as a proxy for cognitive impairment since cognitive impairment and early Alzheimer's disease and related disorders (ADRD) are often unmeasured in administrative data (Figure 1). For more on the concept of proxy confounder adjustment see VanderWeele^3^ and Schneeweiss.^4^

While researcher‐specified confounders are identified using expert background knowledge, empirical or proxy confounders are identified using empirical associations and coding patterns observed in the data. There is a growing body of evidence showing that complementing researcher‐specified variables with empirically‐identified proxy confounders improves confounding control compared to adjustment based on researcher‐specified confounders alone.^4^, ^6^, ^7^, ^8^, ^9^ Consequently, there has been a recent focus on the development of data‐driven methods to empirically identify high‐dimensional sets of proxy variables for adjustment in healthcare database studies.^6^, ^10^, ^11^, ^12^, ^13^, ^14^, ^15^, ^16^, ^17^, ^18^

**FIGURE 2:**
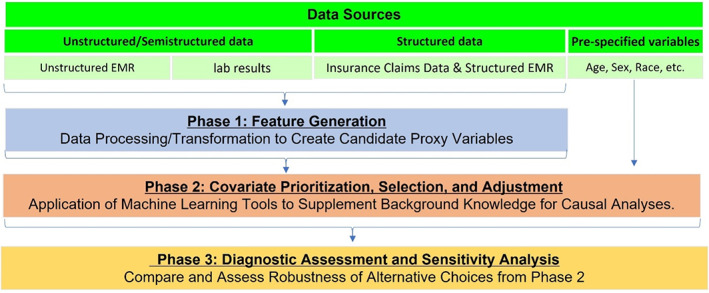
Different phases for high‐dimensional proxy confounder adjustment.

In this paper, we discuss the considerations underpinning three areas for data‐driven high‐dimensional proxy confounder adjustment: (1) feature generation—transforming raw data into covariates (or features) to be used for proxy adjustment; (2) covariate prioritization, selection and adjustment; and (3) diagnostic assessment (Figure 2). We review current approaches and recent advancements within each area, including the most widely used approach to proxy confounder adjustment in healthcare database studies (the high‐dimensional propensity score or hdPS). We discuss limitations of the hdPS and survey recent advancements that incorporate the principles of proxy adjustment with machine learning (ML) extensions to improve performance. We further discuss challenges and directions for future development within each area. We give particular focus to diagnostic assessment for causal inference as this has received the least attention when performing high‐dimensional proxy confounder adjustment in the pharmacoepidemiology literature.

## 2 GENERATING FEATURES FOR PROXY CONFOUNDER ADJUSTMENT

The first challenge for proxy confounder adjustment is determining how to best leverage the full information content in healthcare databases to generate features (or proxy variables) that best capture confounder information. Several approaches for feature generation of proxy confounders have been applied in the pharmacoepidemiologic literature. These have ranged from very simple approaches that generate binary indicators representing whether or not a given code occurs during a pre‐defined exposure assessment period,^19^ to approaches that first process information from healthcare databases into a common data model format with common terminologies and coding schemes representing health concepts.^20^, ^21^, ^22^ Feature engineering can then be applied to the common data model to enable a consistent process across different databases. Examples include the Observational Medical Outcomes Partnership (OMOP) Common Data Model, maintained by the open‐science Observational Health Data Sciences and Informatics (OHDSI) network and also used in the European Health Data and Evidence Network (EHDEN) project, and the National Patient‐Centered Clinical Research Network (PCORnet).^23^, ^24^, ^25^ Generating features consistent with a common data model format can be advantageous for capturing relevant health concepts, but these approaches require more data pre‐processing to extract and transform the original codes into variables representing health concepts.

Instead of generating features based on health concepts, an alternative approach is to generate features based on empirical associations and longitudinal coding patterns observed in the data. Such approaches can be more flexible since they can be independent of the coding system and do not rely on a common data model.^6^ The hdPS has become the most widely used tool to generate features based on observed coding patterns in healthcare claims databases.^6^ The hdPS generates features by transforming raw medical codes into binary indicator variables based on the frequency of occurrence of each code during a defined pre‐exposure period.

By taking into account the frequency of occurrence of various codes during the covariate assessment period, the hdPS tries to capture information on the intensity of the medical event or drug dispensing. In theory, algorithms could consider more complex longitudinal coding patterns to try and capture additional confounder information. For example, recent work has proposed using neural networks to model a patient's full course of care to consider temporal sequences of a specific course of treatment.^26^ The use of neural networks for extracting confounder information by modeling complex coding patterns is promising but examples are limited.^27^, ^28^

### 2.1 Challenges in generating features for proxy adjustment from electronic health records

An important limitation of current high‐dimensional proxy confounder adjustment approaches is that they can only use structured electronic healthcare information. However, much of the essential confounder information, such as patient‐reported symptoms, severity, stage and prognosis of the disease, and functional status, is frequently recorded in free‐text notes or reports in electronic health records (EHRs) that are substantially underutilized for confounding adjustment.^29^, ^30^ Little is known about the impact of incorporating these data for confounding adjustment since unstructured data are not readily analyzable. Natural language processing (NLP) is a subfield of machine learning that can be used to generate variables from unstructured free text.^31^ NLP methods are increasingly used to identify health outcomes from EHRs, but the application of NLP algorithms for purposes of identifying high‐dimensional sets of confounding factors is limited.^32^ More research is needed on the use of NLP algorithms for generating high‐dimensional sets of proxy confounders and the value of unstructured EHR data in proxy adjustment.

An additional challenge to utilizing EHR data for high‐dimensional confounding control is missing data. While both healthcare claims and EHR data are susceptible to missing information, EHR data is particularly vulnerable due to a lack of continuity and completeness of health records caused by patients seeking care at different delivery systems.^33^, ^34^ Various approaches for handling missing data have been proposed, including several alternative multiple imputation techniques. Multiple imputation can account for informative missingness under certain untestable assumptions. However, there are many different approaches to handling missing data and no single approach is universally best.^35^ Failing to appropriately account for missingness and measurement error when using EHR data can result in analyses that increase rather than reduce bias in estimated treatment effects.^36^, ^37^, ^38^

## 3 COVARIATE PRIORITIZATION, SELECTION, AND ADJUSTMENT

Once proxy variables have been generated through transformations of the raw data, some degree of dimension reduction is needed to prioritize and select variables for adjustment. Reducing the dimension of covariates is necessary to avoid problems of nonoverlap when adjusting for high‐dimensional sets of covariates.^39^ Nonoverlap can result in non‐convergence due to separation when sufficiently many covariates are included in case of logistic regression models.^40^ Positivity violations are also a concern for hdPS analyses, as covariate overlap is more difficult to satisfy when controlling for high‐dimensional sets of variables.^39^ Even when the sample size may be large enough to effectively preclude problems related to convergence and positivity, it is not practical to consider every possible adjustment set for high‐dimensional data. Machine learning can help researchers reduce the dimension of covariates to avoid issues of nonoverlap and can more flexibly model selected covariates when predicting the treatment and outcome mechanisms.

### 3.1 hdPS prioritization and its limitations

**FIGURE 3:**
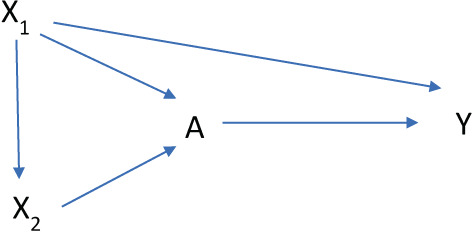
Causal diagram illustrating one scenario where the use of marginal empirical associations for confounder selection can result in over‐adjusting for instrumental variables. In this causal structure, *X*_2_ is marginally associated with both treatment and outcome, but is independent of the outcome after conditioning on *X*_1_.

The hdPS has been the most widely used data‐driven tool in the pharmacoepidemiologic literature for high‐dimensional confounder selection. The hdPS prioritizes or ranks each generated variable based on its potential for bias by assessing the variable's prevalence and univariate, or marginal, association with the treatment and outcome using the Bross bias formula.^6^, ^41^, ^42^ From this ordered list, researchers then specify the number of variables to include for adjustment along with pre‐specified variables. While the hdPS has been shown to often improve confounding control when used to complement investigator‐specified confounders,^6^, ^8^, ^9^, ^43^, ^44^ there are cases where adjustment for hdPS generated variables had no impact or even harmed the properties of estimators beyond adjustment for researcher‐specified confounders alone.^45^, ^46^ Limitations of the hdPS prioritization include: (1) the method assesses a variable's potential confounding impact through marginal, or univariate, associations with both treatment and outcome (ideally one would want to consider conditional, or joint, associations among variables); (2) the method requires researchers to subjectively determine how many “proxy” variables to include for adjustment. These limitations can lead to “*over adjusting*” for variables that can harm the properties of estimators without reducing bias (Figure 3).^47^, ^48^, ^49^ Under adjusting by failing to control for proxy variables that contain important confounder information can also be a concern when implementing the hdPS.

The choice of the number of proxy variables to include in an hdPS model to adequately control for confounding without “*over adjusting*” or “*under adjusting*” varies according to the properties and structure of a given dataset and cannot be identified by only evaluating marginal associations between variables. Determining how many empirically identified “proxy” confounders to include for adjustment is particularly challenging in studies with rare events — settings relevant to RWE studies. In these settings, previous work has shown unstable effect estimates where results are highly dependent on the number of “proxy” confounders included for adjustment.^9^, ^43^

### 3.2 Machine learning extensions for covariate prioritization and selection

To address the limitations outlined above, recent studies have developed extensions for proxy confounder adjustment that combine the principles of proxy confounder adjustment with ML tools for prediction modeling and variable selection. These tools have largely focused on incorporating principles for proxy confounder adjustment with regularized regression and Targeted Learning tools, including Super Learning and Collaborative Targeted variable selection. While other ML tools for variable prioritization and selection are available (e.g., principal components, random forests, feature importance selection with neural networks), here we focus on targeted learning tools and regularized regression as these have been the most widely used approaches in the pharmacoepidemiology literature.

#### 3.2.1 Regularized regression for high‐dimensional proxy confounder adjustment

Regularized regression models use penalized maximum likelihood estimation to shrink imprecise model coefficients toward zero. LASSO is the most commonly used regularized regression model for variable selection in high‐dimensional covariate datasets.^44^, ^50^ Previous work^20^, ^21^, ^22^, ^51^ found that LASSO regression can be used to select a subset of generated “proxy” confounders to supplement researcher‐specified confounders to form the adjustment set for confounding control. To improve the performance of regularized regression for high‐dimensional confounder selection, several studies have developed variations of LASSO that consider covariate associations with both treatment and outcome when penalizing the likelihood function. These recent extensions include: (1) Outcome adaptive LASSO,^17^ (2) Group LASSO,^16^ (3) Highly Adaptive LASSO,^52^ (4) Highly Adaptive Outcome LASSO,^11^ and (5) Collaborative Controlled LASSO.^53^ Other versions of regularized regression, including ridge regression and elastic net, have also been shown to perform well for confounder selection and can be preferable to the LASSO penalization in certain settings.^51^

#### 3.2.2 Combining the hdPS with super learning

Super Learning is an ensemble ML algorithm for prediction modeling that forms a set of predicted values based on the optimal weighted combination of a set of user‐specified prediction models in terms of minimizing cross validated predictive performance.^54^, ^55^ The flexibility of *super learning* can be utilized to identify a small number of optimally performing prediction algorithms that generally perform best for a given data structure. Previous work has combined Super Learning with proxy confounder adjustment in high‐dimensional covariate spaces.^18^ Super Learning can simplify model selection for propensity score estimation in high dimensions and has been shown to perform well in a number of simulations.^13^, ^18^

#### 3.2.3 High‐dimensional proxy adjustment with scalable versions of collaborative targeted maximum likelihood estimation

Collaborative targeted maximum likelihood estimation (CTMLE) is an extension of the doubly robust targeted maximum likelihood estimation (TMLE) method.^56^, ^57^ TMLE consists of fitting an initial outcome model to predict the counterfactual outcomes for each individual, then using the estimated propensity score to fluctuate this initial estimate to optimize a bias/variance tradeoff for a specified causal parameter (i.e., the treatment effect). CTMLE extends TMLE by using an iterative forward selection process to construct a series of TMLE estimators, where each successive TMLE estimator controls for one additional variable to consider how a variable relates to both treatment and outcome after conditioning on a set of previously selected variables.^56^, ^57^ By taking into account a variable's conditional association with both treatment and outcome, CTMLE avoids “*over‐adjustment*” to improve the properties of estimators by reducing the likelihood of controlling for variables that are conditionally independent of the outcome after adjusting for a set of previously identified confounders. Recent work has developed adaptations of CTMLE that are computationally scalable to large healthcare databases.^14^ These adaptations modify the standard version of CTMLE by including a pre‐ordering of variables to avoid the iterative process of searching through each variable in the selection procedure. Simulations indicate that computational gains are substantial and that combining scalable CTMLE with methods for proxy adjustment work well relative to the standard instantiations of CTMLE, hdPS, and TMLE.^14^, ^18^

### 3.3 Adjustment for proxy confounders

Once proxy confounders have been prioritized and selected, researchers must determine a method for adjustment and causal estimation. Propensity score methods (e.g., propensity score matching, inverse probability weighting) using logistic regression for estimation of the propensity score function have become the most common approach for adjustment of selected proxy confounders in the medical literature.^58^, ^59^ Some evidence suggests that improvements can be gained in both predictive performance and bias reduction when using more flexible ML models for propensity score estimation.^28^, ^60^, ^61^, ^62^ Another avenue for improving estimations is to adapt ML algorithms to casual inference. Two important examples are the adaptation of random forest to *causal forest* and *X‐learner*, a meta‐algorithm that uses ML methods as an intermediate step in an efficient estimation algorithm.^63^, ^64^

#### 3.3.1 Machine learning with doubly robust estimation for improved adjustment

Widely used doubly robust methods include TMLE, augmented inverse probability weighting (AIPW), and double ML (e.g., R‐learner).^57^, ^65^, ^66^, ^67^ These approaches use a model for both the outcome and the propensity score, requiring only one of the two to be correctly specified for consistent estimation of average treatment effects. Theory and simulations have shown that doubly robust approaches are asymptotically efficient and more robust than conventional singly robust methods like propensity score matching and inverse probability weighting.^68^

Recent work has further shown that the use of flexible nonparametric ML models for the estimation of nuisance functions (i.e., the propensity score or outcome model) comes at a cost of slow convergence rates. This slow convergence is particularly problematic within singly robust estimation methods and can yield effect estimates with poor statistical properties with performance deteriorating as the dimension of the data increases (the ‘curse of dimensionality’).^69^ This work has further demonstrated that doubly robust methods allow for slower converging nuisance models and, therefore, can mitigate or even resolve such problems. Consequently, recent literature suggests that ML‐based methods for estimation of nuisance functions should be applied within doubly robust frameworks rather than more commonly used singly robust methods. For more on machine learning in causal inference see Kennedy,^69^ Naimi et al.,^70^, ^71^ and Zivich et al.^72^

## 4 DIAGNOSTIC VALIDITY ASSESSMENT OF CAUSAL ESTIMATIONS

Evaluating the validity of causal analyses for high‐dimensional proxy adjustment remains challenging but is essential to improving robustness and validity of estimated effects.^73^ While held‐out sets and cross‐validation allow a direct comparison of ML predictions to observed target variables, such a straightforward evaluation is infeasible in causal inference and the role of prediction diagnostics for purposes of causal inference is less clear.^47^, ^74^, ^75^, ^76^ Below, we survey a list of standard ML diagnostics for model prediction and diagnostics for causal inference with a focus on assessing the performance of models for high‐dimensional proxy adjustment in their ability to reduce bias in estimated treatment effects. We highlight their underlying assumptions and limitations.

### 4.1 Diagnostics for treatment and outcome model prediction

A process to estimate ML model performance using out‐of‐sample data, such as cross validation, are often recommended to assess model robustness and generalizability and to examine the characteristics of the inferred models to verify the importance of domain‐relevant variables. Below we focus on additional measures with specific importance to causal model diagnostics.

#### 4.1.1 Dichotomous and categorical models

*Calibration plots* depict the average predicted versus observed (empirical) probability of the studied event in subsets of entities (typically, deciles), to evaluate the accuracy of the predicted probabilities.^77^, ^78^ Probability estimation accuracy is essential for causal inference, more than it typically is for ML classification tasks, as downstream calculations, for example, inverse probability weighting, may rely on these values as being “true” probabilities. Various metrics can be used to quantitatively measure calibration quality, for example, Hosmer‐Lemeshow goodness of fit test,^79^ but these have several drawbacks^80^; visual inspection of the calibration plots or characterization of its slope and intercept is thus recommended.

*C‐statistic* (or area under the receiver operating characteristic, ROC, curve), a measure of classification accuracy, is commonly used in standard ML applications. For outcome models, it can be used to assess prediction accuracy over the observed treatment assignment (and assuming, but not verifying, that the causal assumptions hold). For propensity models its utility is less straightforward: an extreme (close to 0 or 1) value, corresponding to a highly discriminative model, may indicate a potential violation of positivity; and, conversely, a value around 0.5, suggesting the model cannot discriminate between treatment groups, is not necessarily a sign for inaccurate model, but potentially good covariate overlap. As a result, some researchers recommended to avoid using C‐statistic in propensity model diagnostics.^75^ We note that post‐matching C‐statistic may be used to evaluate covariate balance; see below.

#### 4.1.2 Continuous models

The performance of continuous outcome models can be assessed in each observed treatment group (and observed outcomes) and assuming causal assumptions are met, using standard measures such as the coefficient of determination (*R* ^2^) or mean squared error.^78^ A poorly performing model for a specific treatment group, for example, over or underestimating outcomes, may subsequently lead to biased effect estimation. As with binary outcome models, poor performance may suggest an inadequate prediction model and guide its improvement.

### 4.2 Diagnostics for causal inference

Previous work has shown that the use of prediction model diagnostics alone to guide model selection and validity assessment can lead to suboptimal performance for causal inference.^47^, ^48^, ^49^, ^75^, ^81^ We next survey diagnostic methods to more directly assess assumptions and model validity for purposes of causal inference.

#### 4.2.1 Positivity

An important usage for propensity models for high‐dimensional proxy adjustment is to examine the positivity assumption. This assumption states that every individual has a non‐zero probability to be assigned to any treatment conditional on a sufficient set of covariates. A comparison of propensity score distributions can help in identifying (and potentially excluding) sub‐populations where violations or near violations of the positivity assumption occur.^82^, ^83^, ^84^ While high‐dimensional proxy adjustment assumes that unconfounded treatment effects are more plausible when controlling for large numbers of variables, covariate overlap can be more difficult when adjusting for high‐dimensional sets of variables.^39^ Therefore, positivity should be tested at the initial stages of analyses for high‐dimensional proxy adjustment.

#### 4.2.2 Balancing

Propensity score modeling aims to facilitate matching, reweighting or stratification to emulate a random assignment of individuals to treatment groups. Therefore, several studies explored methods to directly evaluate balancing of covariates among these groups.^77^, ^78^, ^85^ In a simulation study, Franklin et al.^85^ compared several metrics to assess covariate balance and observed that two had consistently strong associations with bias in estimated treatment effects. The first metric, post‐matching C‐statistic, re‐trains a treatment model on the propensity score matched (similarly, stratified or weighted) sample and assesses its (preferably, lack of) ability to discriminate between patients in different treatment groups using C‐statistic. The second recommended metric, general weighted difference, computes a weighted sum of absolute difference in all individual covariates, all covariate squares, and all pairwise interactions. Other papers have also recommended assessing the standardized mean difference in covariates for PS matching and weighting.^86^, ^87^

The application of balance diagnostics for high‐dimensional propensity scores is more challenging as it is unclear on which set of variables balance should be assessed. A large literature has shown that balancing variables that are independent of the outcome except through treatment (instrumental variables) harms the properties of estimators.^47^, ^49^, ^81^ In high‐dimensional settings, however, identifying instrumental variables is difficult and previous work has argued that priority should be given to controlling for all confounders at the expense of balancing instruments.^20^, ^21^, ^48^ This has led to some researchers assessing balance on all variables in the database when using propensity scores for high‐dimensional proxy adjustment.^20^, ^21^, ^22^ More research is needed on the best use of balance diagnostics for high‐dimensional propensity score adjustment.

#### 4.2.3 Estimand diagnostics (simulation‐based approaches and negative controls)

Recent studies have suggested methods to assess the overall accuracy of effect estimation using control and synthetic control studies.^20^, ^21^, ^88^, ^89^, ^90^ These frameworks have largely been based on the use of simulation methods to generate synthetic datasets under constraints where certain relations among variables are known (e.g., the simulated treatment effect) while maintaining much of the complexity and statistical properties of the observed data structure.

##### Parametric bootstrap (‘Plasmode’ simulation)

Simulation frameworks for model validation in causal inference have largely been based on use of the parametric bootstrap. Such approaches bootstrap subjects from the observed data structure, then use modeled relationships from the original data to inject causal relations between a subset of variables while leaving all other associations among variables unchanged. With treatment‐outcome associations known by design and patterns of confounding that mimic the observed data structure, synthetic datasets have become increasingly popular to provide a benchmark for comparing statistical methods for causal inference.

Franklin et al.^89^ proposed using a parametric bootstrap approach, termed ‘plasmode simulation’, to compare causal inference methods in settings specific to healthcare database studies and high‐dimensional propensity scores. Schuler et al.^90^ and others^88^, ^91^, ^92^ have proposed variations and extensions of plasmode simulation for model validation in healthcare database studies. Schuemie et al.^20^, ^21^ use a plasmode simulation‐based approach for generating positive control outcomes to quantify bias due to measured confounders when calibrating effect estimates and confidence intervals. Peterson et al.^93^ apply a similar parametric bootstrap method as a diagnostic to assess bias due to violations of positivity. Alaa and van der Schaar^88^ developed a validation method that uses the parametric bootstrap and influence functions, which are a key technique in robust statistics.

While simulations can be useful for tailoring analytic choices for causal inference, they also have limitations that deserve attention. Schuler et al.^90^ explain that validation frameworks based on the parametric bootstrap are more limited since they are not ‘model free’; they require partial simulation of the data structure. This creates two fundamental challenges when generating synthetic datasets to evaluate causal inference methods: (1) Advani et al.^94^ showed that if the simulation framework does not closely approximate the true data generating distribution, then the use of synthetically generated data as a diagnostic tool in causal inference can be misleading; (2) even when the simulation framework closely approximates the true data generating process, Schuler et al.^90^ warn that the use of synthetic datasets for model validation could still be biased towards favoring causal inference methods that mimic the modeling choices made when generating the synthetic datasets. These challenges can restrict the usefulness of synthetic datasets for model validation in causal inference. Still, studies have demonstrated that in specific cases, the use of synthetic data to tailor analyses to the study at hand can often improve confounding control relative to the consistent use of any single causal inference method.^88^, ^90^

*Wasserstein Generative Adversarial Networks (WGANs)* is an alternative approach to generating synthetic data for simulation‐based model validation in causal inference.^95^ GANs estimate the distribution of a particular dataset using a ‘generator’ and a ‘discriminator’.^96^ The generator is a flexible neural network to create synthetic data while the discriminator is a competing neural network model that attempts to distinguish between the synthetic and real data. The process is repeated in an iterative fashion until the discriminator is no longer able to distinguish between the synthetic and real data. This technique has become very powerful for supervised and unsupervised ML.^96^ WGANS have recently been shown to be useful for generating synthetic datasets that closely approximate the joint correlation structure of an actual dataset for purposes of model validation in causal inference.^95^

##### Negative and positive controls

Another approach that has become increasingly popular for evaluating models for confounder adjustment is the use of real negative controls—exposure‐outcome pairs that are not, as far as we know, causally related.^77^, ^97^ Such controls can be used to detect residual biases, for example, confounding, in the estimation process. Replicating a known association through use of positive controls can also increase confidence in primary estimates' validity. However, some researchers have argued that identifying positive controls is difficult since the magnitude of known effects is rarely known.^20^, ^21^, ^22^

#### 4.2.4 Sensitivity analyses

##### Quantitative bias analysis

Estimating an effect from observational data involves multiple, at times somewhat arbitrary, modeling decisions and assumptions, for example, with respect to the definition of confounders, exposures, and outcomes or the statistical analysis.^98^ Sensitivity analysis re‐computes the estimated effect under various sets of such decisions^99^ or using multiple data sources to verify its robustness.^83^, ^100^ Sensitivity analyses can also quantify the change that an unmeasured confounder would have on the studied estimand and thus assess its sensitivity to violations of the assumption of no unmeasured confounding.^99^, ^101^ This can be particularly useful when applying methods for high‐dimensional proxy adjustment as researchers can never be certain how well a set of features captures information on unmeasured factors. The E‐value in particular has become widely used for assessing sensitivity of an estimand to unmeasured confounding in the medical literature.^102^ The popularity of the E‐value has largely been due to its simplicity, making its implementation and communication straightforward. However, its simplicity has also been a point of criticism of the method.^103^, ^104^, ^105^, ^106^, ^107^ Several more comprehensive bias analysis methods have been developed to quantify the impact of various systematic errors to increase confidence that the estimated effects are robust to violations of various assumptions. Lash et al. provide a detailed discussion on methods for quantitative bias analysis.^108^ An overview of a subset of diagnostics for causal inference is shown in Table 1.

**TABLE 1:** Examples of diagnostic metrics for causal inference.

Condition being tested	Possible diagnostic checks	Limitations and comments
Positivity	Overlap of estimated PS across treatment groups	Impact of limited overlap can depend on the adjustment approach Including more covariates for adjustment can decrease overlap. Consequently, it can be difficult to determine the optimal adjustment set in terms of maximizing confounding control vs bias due to nonoverlap
Conditional Exchangeability on Measured Covariates	Covariate balance across treatment groups after PS adjustment	Primarily used for PS analyses. Less useful for causal inference approaches that model that outcome directly, including doubly robust methods. Can be difficult to quantify the impact of residual imbalance on bias in estimated treatment effects Can be difficult to determine on which variables balance should be assessed (e.g., do not want to balance instrumental variables).
	Prediction diagnostics to assess correct model specification	Can reward PS models that include instruments More useful for causal inference approaches that model the outcome, including doubly robust methods
	Simulation‐based approaches for generating synthetic datasets to evaluate bias in estimated treatment effects	A very general approach that is applicable to any causal inference method Requires advanced simulation techniques to closely approximate the confounding structure of the study population
Violation of conditional exchangeability due to unmeasured confounding	Real negative and positive control exposures and/or outcomes	Can be useful to identifying bias caused by unmeasured confounders Can be difficult to identify good negative and/or positive controls
Sensitivity to hidden biases (e.g., unmeasured confounding, misclassification)	E‐value	Implementation and communication is simple and straightforward Recent critiques have argued that the E‐value can be misleading due to its simplicity
	Formal quantitative bias analysis	Several approaches have been proposed to conduct in‐depth sensitivity analyses for hidden biases. These can provide more detailed assessment of robustness of causal analyses, but are subject to underlying assumptions and can be tedious to implement

## 5 DISCUSSION

In this paper, we have provided an overview of high‐dimensional proxy confounder adjustment in studies utilizing electronic healthcare databases. We have focused on three areas for proxy adjustment: (1) feature generation, (2) covariate prioritization, selection, and adjustment, and (3) validity assessment. We have discussed recent ML extensions and paths for future research within each area. Much attention has been given to the development of ML tools for confounder selection and adjustment for high‐dimensional proxy adjustment. These tools have great potential to improve confounding control in healthcare database studies. However, less attention has been given to advancing methods for feature generation and validity assessment for proxy confounder adjustment. Future research is warranted to investigate the optimal methods that extract the relevant confounding information to generate features for proxy adjustment while preserving scalability and data‐adaptability to large healthcare databases. Future research is also needed in the development of diagnostic methods to evaluate and compare the validity of alternative approaches to high‐dimensional proxy adjustment in healthcare database studies.

Finally, although ML tools can be beneficial in identifying empirical associations among large numbers of covariates, empirical associations by themselves are not sufficient to determine causal relations.^109^, ^110^, ^111^ We emphasize the importance of using substantive knowledge to obtain an understanding of the data and the underlying causal structure before applying ML procedures for confounding control.^109^, ^110^, ^111^ ML procedures should not replace background knowledge, but should be used to complement investigator input when controlling for confounding.

## AUTHOR CONTRIBUTIONS

Richard Wyss, Chen Yanover, and Tal El‐Hay drafted the manuscript. All authors revised the manuscript for important intellectual content and approved the final manuscript to be submitted for publication.

## FUNDING INFORMATION

Funding to support this manuscript development was provided by the International Society for Pharmacoepidemiology (ISPE).

## CONFLICT OF INTEREST

Robert W. Platt has consulted for Amgen, Biogen, Merck, Nant Pharma, and Pfizer. Dimitri Bennett is an employee of Takeda. Grammati Sari is employed by Visible Analytics Ltd. Hongbo Yuan is an employee of CADTH. Andrew R. Zullo receives research grant funding from Sanofi Pasteur to support research on infections and vaccinations in nursing homes unrelated to this manuscript. Mugdha Gokhale is a full‐time employee of Merck and owns stocks in Merck. Elisabetta Patorno is supported by a career development grant K08AG055670 from the National Institute on Aging. She is researcher of a researcher‐initiated grant to the Brigham and Women's Hospital from Boehringer Ingelheim, not directly related to the topic of the submitted work.
